# Comparison of Enriched Acoustic Environment and White Noise as Sound Stimuli for Tinnitus Treatment: A 4-Month Feasibility Study

**DOI:** 10.3390/brainsci15040342

**Published:** 2025-03-26

**Authors:** Marta Fernández-Ledesma, Ricardo Sanz-Fernández, María Cuesta, Pedro Cobo

**Affiliations:** 1 Department of Medicine, Faculty of Biomedical and Health Sciences, European University of Madrid, C/Tajo s/n, 28670 Villaviciosa de Odón, Spain; marta.fernandez2@universidadeuropea.es (M.F.-L.); ricardosanz.orl@gmail.com (R.S.-F.); 2 Institute for Physical and Information Technologies (ITEFI), Spanish National Research Council (CSIC), 28006 Madrid, Spain; m.cuesta@csic.es

**Keywords:** tinnitus, sound therapy, EAE, white noise, HADS, THI, TFI

## Abstract

**Background/Objectives**: This study evaluated the feasibility and effectiveness of three sound therapies—enriched acoustic environment with random noise (EAERR), enriched acoustic environment with gamma tones (EAEGT), and white noise (WN)—in alleviating tinnitus distress and enhancing emotional well-being. **Methods**: A total of 125 individuals with tinnitus were recruited, with 92 completing the four-month intervention. Following counseling, participants selected a therapy and listened daily for one hour for four-months at the mixing point intensity. Tinnitus severity and emotional state were assessed at the baseline and post-treatment using the Tinnitus Handicap Inventory (THI), Tinnitus Functional Index (TFI), and Hospital Anxiety and Depression Scale (HADS). **Results**: All therapies significantly reduced tinnitus distress and improved emotional well-being, with 80.4% of participants reporting benefits. **Conclusions**: These findings suggest that sound therapies are effective for tinnitus management, though further research with larger and more homogeneous samples is needed to refine their application and optimize treatment for diverse tinnitus profiles.

## 1. Introduction

Tinnitus is characterized by the perception of sound without an external auditory stimulus [1] and is estimated to affect approximately 14% of the global population [2]. Despite its prevalence, no pharmacological treatments have been specifically developed for tinnitus. In recent years, sound therapies have emerged as one of the most employed interventions to alleviate tinnitus symptoms and modify patients’ perception or reactions to the condition [3,4,5].

The development of controlled clinical trials to evaluate sound therapies for tinnitus faces significant challenges, primarily due to difficulties in selecting an appropriate “placebo sound” and the influence of individual personality traits on sound preference. As a result, few studies have directly compared various sound therapy modalities for tinnitus treatment under standardized conditions [6]. In light of these limitations, Jastreboff and Hazell [1] suggested that “any sound is better than silence” for tinnitus therapy. Although sound therapy is widely used, there is still no agreement on the ideal sound characteristics for tinnitus treatment, nor is it clear if its benefits surpass those provided by hearing aids or psychological interventions [7,8].

Some studies have found only small or statistically insignificant differences between various sound therapies [9,10]. However, certain individualized treatments tailored to the patient’s specific hearing loss have shown greater effectiveness compared with interventions based solely on counseling, with or without the use of broadband noise [11]. Conversely, evidence from prior research suggests that temporally dynamic sounds may result in greater reductions in tinnitus symptoms compared with fixed-intensity sounds [12]. This phenomenon could be explained by the ability of dynamic sounds to enhance informational (central) auditory masking, effectively creating competition between the therapeutic sound and the tinnitus percept for cognitive processing resources [13].

This study aimed to evaluate the feasibility of three distinct sound stimuli for tinnitus therapy: enriched acoustic environment (EAE) with random noise (EAERR), EAE with gamma tones (EAEGT), and white noise (WN). The EAE as sound therapy has recently been proposed [14,15,16,17] and is implemented either as a continuous random noise (EAERR) or a sequence of gammatones (EAEGT), both filtered by the hearing loss curves of the subject. WN consists of a stereo white noise and is not personalized to the hearing of the subject.

The EAERR is the implementation of the proposal by Schaette and Kempter [18]. Using a computational model, they demonstrated that a stimulus with spectrum matched to the HL curves was able to revert the tinnitus induced hyperactivity produced by homeostatic plasticity.

The EAEGT is a modification of the enriched acoustic environment (EAE) used by Noreña and Eggermont [19] to revert the reorganization of the tonotopic map of cats previously exposed to a traumatizing noise. When these cats were immersed in a sound field consisting of a sequence of tone-pips of random frequency within their hearing band, the tonotopic maps returned to those measured before the traumatizing reorganization. The spectrum of each tone can be controlled by a couple of values (α, γ), which can then be used to match the frequency response of different parts of the auditory system (basilar membrane, auditory neurons) of each specie. For humans, these parameters are determined by the gamma filters. Therefore, the appropriate tone-pips for humans are the gammatones [14]. Another variation of EAEGT with respect to the sequence of tone-pips is that the amplitude of the tones is matched to the HL value at the corresponding frequency [17].

Previous studies indicated that EAE therapy yields more favorable outcomes for tinnitus patients compared with other sound therapies [14,15,16,17,20,21].

## 2. Materials and Methods

### 2.1. Participants

The study received approval from the Research Ethics Committee of the European University of Madrid (protocol code CIPI/23.009 approved 30 January 2023) and was conducted in compliance with the Declaration of Helsinki for research involving human subjects as well as the Spanish Data Protection Law (RD1720/2007).

**Figure 1:**
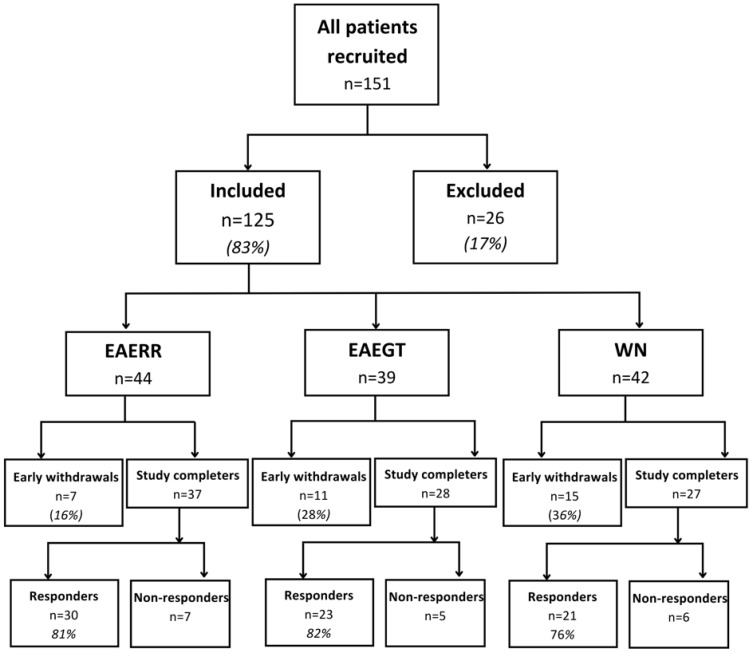
Flow diagram of participants in this study. EAERR: enriched acoustic environment therapy with continuous sound; EAEGT: enriched acoustic environment therapy with sequential sound; WN: white noise.

A total of 151 volunteers with tinnitus (see Figure 1) provided their written informed consent and were recruited through association outreach, advertisements, and word-of-mouth referrals. Participants aged 18 to 80 years with non-pulsatile tinnitus, a depression subscale of the Hospital Anxiety and Depression Scale (HADSD) score below 16, and a Tinnitus Handicap Inventory (THI) score of ≥20 points were included in this study. Patients with recent ear surgery, severe Ménière syndrome, and hydrocephaly were excluded. After applying these criteria, 125 patients (83%) were enrolled and 26 (17%) were excluded.

All participants attended a standardized counseling session addressing the relationship between tinnitus, the limbic system, and the autonomic nervous system. Following this session, the participants selected a preferred auditory stimulus for therapy: 44 participants chose EAERR, of whom 7 (16%) dropped out; 39 selected EAEGT, with 11 (28%) dropping out; and 42 opted for WN, with 15 (36%) dropping out.

Once enrolled in the study, the participants underwent the following assessments: pure tone audiometry (air and bone pathways), discomfort thresholds, tinnitus characterization, and completion of the Tinnitus Handicap Inventory (THI), Tinnitus Functional Index (TFI), and Hospital Anxiety and Depression Scale (HADS) questionnaires. Subsequently, they were invited to select one of three sound stimuli based on their level of comfort. Participants were instructed to listen to their chosen stimulus for one hour daily at an intensity adjusted to the mixing point (just below their tinnitus intensity).

Throughout the four-month intervention, a monthly follow-up was conducted via telephone, and the participants submitted all questionnaires via email. At the end of the fourth month, tinnitus matching and discomfort threshold measurements were repeated.

Out of the 151 recruited participants, 26 participants (17%) were excluded before treatment initiation. Among the 125 included participants, the dropout rates varied across groups: EAERR had the lowest dropout rate (16%, n = 7); EAEGT had a moderate dropout rate (28%, n = 11); WN had the highest dropout rate (36%, n = 15). In most cases, dropouts occurred early in the intervention period, often within the first few therapy sessions. Possible reasons include discomfort with the sound stimulus, a lack of immediate symptom relief, or difficulty adhering to the intervention schedule. The low dropout rate of the EAERR group may be attributed to the fact that it was the stimulus that most people found comfortable. Conversely, the WN group’s higher dropout rate could have stemmed from the less engaging nature of white noise therapy, which some participants may have perceived as less effective, annoying, or monotonous.

### 2.2. Hearing Levels

**Figure 2:**
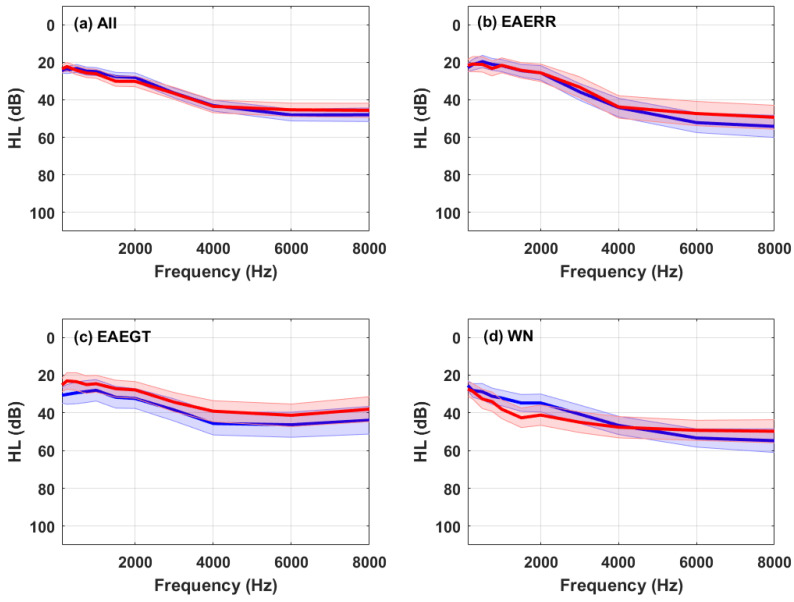
Average right ear (red) and left ear (blue) hearing levels (HLs) of (**a**) all participants who completed the treatment; (**b**) participants who selected EAERR; (**c**) participants who selected EAEGT; and (**d**) participants who selected WN. The shaded area around the curves outlines the confidence intervals (±1.96 SD/sqrt(N)), where SD is the standard deviation and N is the number of subjects.

The EAERR and EAEGT sound therapies were designed to selectively stimulate the auditory system of the participants according to their individual hearing loss profiles. The pure-tone audiometry of each participant was measured in our laboratory, covering frequencies from 125 Hz to 8 kHz. The mean audiometric thresholds (hearing levels (HL)) for all participants and categorized by sound therapy are shown in Figure 2.

### 2.3. Tinnitus Characteristics

Participant case histories were gathered immediately after obtaining informed consent using a standardized tinnitus characteristics questionnaire. The data collected included details on tinnitus attributes such as temporal variability, perceived frequency, sound type, and lateralization (bilateral, left ear, or right ear). Participants also provided information regarding the presumed etiology of their tinnitus, previous treatments, and relevant comorbidities. Among the 74 participants who had improved tinnitus after treatment, 34 (46%) reported bilateral tinnitus, 22 (30%) localized their tinnitus to the left ear, and 18 (24%) to the right ear. Regarding tinnitus type, 45 participants (61%) described their tinnitus as tonal, 22 (30%) reported a hissing sound, and 7 (9%) characterized it as ringing.

Table 1 presents the number of responding subjects who selected each sound therapy, stratified by gender, along with the mean age (M), standard deviation (SD), duration of tinnitus, tinnitus frequency, and initial THI score. Two-sample Kolmogorov–Smirnov tests were applied to assess the null hypothesis that the data in pairs of variables (for instance, age for all-EAERR, all-EAEGT, and all-WN subgroups) were from the same continuous distribution. All tests resulted in the acceptance of the null hypothesis (i.e., all paired vectors came from the same distribution). The corresponding data for the non-responding subjects is presented in Table 2.

### 2.4. Counseling

After providing consent and completing the audiometric testing, participants attended a counseling session lasting approximately 60 min. During this session, a PowerPoint presentation was utilized to provide an overview of the auditory system’s functioning as well as the mechanisms, etiology, epidemiology, and potential treatment options for tinnitus.

### 2.5. Sound Therapy

After the counseling session, the participants chose a stimulus (EAERR, EAEGT or WN) and were provided with detailed instructions regarding its use including guidance on the sound volume, headphone type, and recommended listening schedule. Participants of the three types of therapy used the same hearing protocol: they listened to the chosen stimulus one hour per day for four months. In all cases, the stimuli were administered to the subjects as an audio file, in mp3 format, so that they played them with an audio device (smart phone, tablet, mp3 player, etc.) connected to headphones at a volume just below their tinnitus intensity (mixing point).

### 2.6. Tinnitus Severity

Tinnitus severity was evaluated using the Spanish versions of the THI [22] and TFI [23] questionnaires. Both questionnaires were sent to the participants, along with the HADS. The THI comprises 25 items divided into functional, emotional, and catastrophic subscales, with responses scored as “yes” (4 points), “sometimes” (2 points), or “no” (0 points). The total score ranges from 0 to 100, with severity classified as slight (0–16), mild (18–36), moderate (38–56), severe (58–76), or catastrophic (78–100). To achieve a clinically relevant change, it must decrease at least 7 or 20 points according to the new [24] or the old [25] criteria.

The TFI consists of 25 items across eight subscales: intrusiveness, emotions, cognition, auditory difficulties, sleep, quality of life, energy, and relaxation. Responses range from 0 (never) to 10 (always), and the total score, obtained by averaging the responses and multiplying by 10, ranges from 0 to 100. TFI scores are categorized as: not a problem (0–17), small problem (18–31), moderate problem (32–53), big problem (54–72), or very big problem (73–100). To achieve a clinically relevant change, it must decrease at least 13–14 points [8,26,27].

### 2.7. Emotional State

The emotional state of the participants was assessed using the Spanish version of the HADS [28,29]. Participants completed the questionnaire sent by email, along with instructions for completion. The HADS is commonly used in clinical and research settings due to its brevity and ease of use, requiring no face-to-face interview. The scale consists of 14 items, split into two subscales: anxiety (HADSA) and depression (HADSD). Each item has four response options, scored from 0 (not at all) to 3 (very often). HADSA evaluates symptoms of nervousness, tension, and worry, while HADSD measures low mood, hopelessness, and loss of interest. Each subscale score ranges from 0 to 21, with scores ≥ 8 indicating clinically significant symptoms and scores ≥ 11 indicating moderate to severe disorder.

## 3. Results

Tinnitus relief was quantified using the THI and TFI tools, designed to assess the distress associated with tinnitus. The effectiveness of the therapy was determined by evaluating the change in THI and TFI scores over the four-month intervention period. As described in Figure 1, 92 out of 151 participants (61%) completed the full four-month sound therapy regimen. Of these, 74 participants (80.4%) exhibited a reduction in tinnitus-related distress (responders), while 18 participants (19.6%) showed no significant improvement (non-responders).

The mean score changes, represented as ΔQuest = Quest_baseline_ − Quest_final_, where Quest is the particular questionnaire (THI, TFI of HADS) for the responding participants, are presented in Table 3. All observed ΔTHI and ΔTFI exceeded the 15% improvement in the total scale range, a rule of thumb recently suggested as the minimal clinically important difference [24]. Furthermore, all of them were statistically significant, as all *p*-values were less than 0.001. Regarding HADSA and HADSD, all observed reductions were regarded as clinically relevant [30,31]. Furthermore, all of them were statistically significant, except for the HADSD for WN. The corresponding data for the non-responding participants are summarized in Table 4. In this case, the results were not statistically significant as all *p*-values were greater than 0.05.

**Figure 3:**
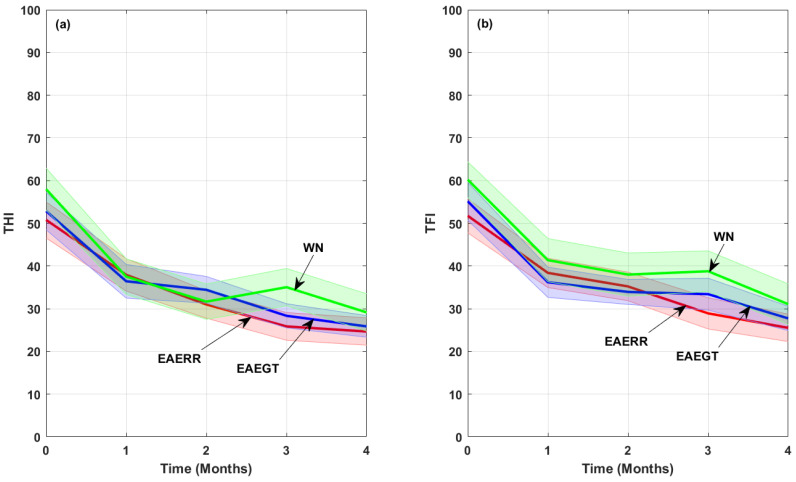
Mean reduction in (**a**) THI and (**b**) TFI scores for the participants for each sound therapy. The shaded area around the curves outlines the confidence intervals (±1.96 SD/sqrt(N)), where SD is the standard deviation and N is the number of subjects.

Figure 3 illustrates the reduction in tinnitus severity in both the THI and TFI questionnaires for each sound therapy subgroup. From Table 2 and Figure 3, it can be inferred that both questionnaires provided almost identical results as evaluators of the tinnitus severity at the baseline and were equally able to detect changes related to the application of the sound treatment [16].

## 4. Discussion

This study evaluated the feasibility and effectiveness of three sound therapies—EAERR, EAEGT, and WN—for reducing tinnitus severity and improving emotional well-being over a four-month intervention with daily one-hour sessions. All therapies significantly reduced tinnitus-related distress (THI, TFI) and improved their emotional state (HADSD and HADSA).

The results are noteworthy as these short daily sessions achieved outcomes comparable to or exceeding those of traditional tinnitus retraining therapy (TRT), which requires continuous sound exposure [25,32]. This is particularly relevant given that our approach involves only one hour per day, which may improve treatment adherence as it does not require hearing aids and can be carried out with regular headphones. Furthermore, patients are encouraged to engage in an activity that maintains their attention, which, as suggested by Kidd et al. [13], may optimize cognitive resource allocation. Shorter therapy sessions could also allow individuals to practice attention techniques, which previous studies have linked to reductions in tinnitus severity [33,34].

Although all therapies demonstrated clinical effectiveness, WN showed slightly greater average improvements. However, this may reflect the higher baseline severity in the WN group (THI: 58, TFI: 60.2) compared with EAERR (THI: 50.8, TFI: 51.8) and EAEGT (THI: 52.9, TFI: 55.2). The potential for improvement in WN was higher but resulted in smaller-than-expected differences in the final outcomes across therapies. For THI, the improvements were Δ(WN-EAERR) = 2.8 and Δ(WN-EAEGT) = 1.9, while for TFI, Δ(WN-EAERR) = 3 and Δ(WN-EAEGT) = 1.8. Additionally, WN exhibited a higher percentage of dropouts (36%) and not improving (24%) compared with the personalized therapies, suggesting that those who completed the therapy were, for the most part, individuals who perceived considerable improvement during the process. Since the calculations only accounted for those who completed the treatment, the mean improvements may have been inflated due to the early dropout of individuals with less progress or no improvement. As WN appears to be a stimulus that encourages dropout, focusing solely on those who improved could introduce a bias in its favor, potentially overestimating its true effectiveness.

Furthermore, we should take into account that the proportional composition of severity grades in each subgroup was different, and the participants with higher baseline scores tended to show a greater reduction [17]. The distribution of tinnitus severity levels based on THI scores varied across the three intervention subgroups: EAERR, EAEGT, and WN. In the EAERR subgroup (30 participants), 37% had mild tinnitus (mean ΔTHI = 15.6), 23% had moderate tinnitus (mean ΔTHI = 25.4), 23% had severe tinnitus (mean ΔTHI = 35.1), and 17% had catastrophic tinnitus (M = 37.6). In the EAEGT subgroup (23 participants), 22% had mild tinnitus (mean ΔTHI = 15.6), 39% had moderate tinnitus (mean ΔTHI = 17.6), 26% had severe tinnitus (mean ΔTHI = 35.3), and 13% had catastrophic tinnitus (mean ΔTHI = 57.6). In the WN subgroup (21 participants), 24% had mild tinnitus (mean ΔTHI = 12.4), 24% had moderate tinnitus (mean ΔTHI = 30.8), 28% had severe tinnitus (mean ΔTHI = 34.7), and 24% had catastrophic tinnitus (mean ΔTHI = 36.4).

In a similar way, the distribution of participants across the TFI severity levels were different in each therapy subgroup. In the EAERR subgroup, 17% had mild tinnitus (mean ΔTFI = 10), 33% had moderate tinnitus (mean ΔTFI = 24.8), 33% had severe tinnitus (mean ΔTFI = 33.2), and 17% had catastrophic tinnitus (mean ΔTFI = 31.4). In the EAEGT subgroup, 8% had mild tinnitus (mean ΔTFI = 7.2), 48% had moderate tinnitus (mean ΔTFI = 19.8), 22% had severe tinnitus (mean ΔTFI = 26.6), and 22% had catastrophic tinnitus (mean ΔTFI = 53.2). In the WN subgroup, 5% had mild tinnitus (mean ΔTFI = 22.4), 29% had moderate tinnitus (mean ΔTFI = 24.5), 38% had severe tinnitus (mean ΔTFI = 27.8), and 29% had catastrophic tinnitus (mean ΔTFI = 36.8).

Baseline differences in hearing loss profiles may also have influenced the outcomes. The WN subgroup exhibited greater hearing loss, particularly at lower frequencies (see Figure 2), which may explain its higher initial scores and the preference for WN. Despite its perceived comfort, tinnitus tends to be more bothersome in individuals with severe hearing loss, complicating masking efforts.

Adherence varied notably across subgroups, with dropout rates of 36% for WN, 28% for EAEGT, and 16% for EAERR. Initial anxiety levels were lowest in the WN subgroup (HADSA = 10.1) compared with EAERR (HADSA = 11.4) and EAEGT (HADSA = 10.6). The final reduction was also smallest in WN (ΔHADSA = 3.6) compared with the personalized therapies, EAERR (ΔHADSA = 5.5) and EAEGT (ΔHADSA = 4.9).

This study provides valuable insights into the potential of shorter sound therapies with various stimuli to achieve outcomes comparable to, or even surpassing conventional treatments. However, several limitations should be noted, which include unequal sample sizes across groups, variability in hearing loss severity, differences in baseline tinnitus severity, and heterogeneity in psychiatric profiles. As a feasibility study, these findings serve as an initial reference for future controlled research, where standardized protocols will be implemented to address these limitations and enhance methodological rigor.

## 5. Conclusions

This study demonstrated that sound therapies, including EAERR, EAEGT, and WN, effectively reduce tinnitus distress and enhance emotional well-being. Over the four-month intervention, 80.4% of participants experienced clinically significant improvements in tinnitus-related distress (THI, TFI) and emotional state (HADSA, HADSD). While WN showed slightly greater improvements, this difference likely reflects the group’s higher baseline severity and hearing loss rather than the therapy’s inherent superiority.

However, WN also had higher dropout and worsening rates compared with personalized therapies, suggesting that those who remained in treatment were primarily individuals who experienced meaningful improvements. Since only completers were analyzed, the findings may overestimate WN’s effectiveness by excluding those who discontinued early due to limited or no progress.

Notably, adherence was highest in the EAERR group, indicating that therapy selection may be influenced by factors such as comfort and ease of use rather than efficacy alone. While all therapies resulted in clinically meaningful symptom reductions, differences in baseline severity, hearing loss, dropout rates, and lack of improvement likely influenced the individual outcomes, underscoring the importance of personalized treatment approaches.

Future research should focus on larger, more homogeneous samples to refine the comparative assessment of sound therapies. Additionally, exploring individual differences, including tinnitus characteristics and psychological factors, will be essential in optimizing treatment strategies for diverse tinnitus profiles.

## Figures and Tables

**Table 1:** Number of responding subjects in each sound therapy group along with the gender, age, tinnitus duration, tinnitus frequency, and initial THI score. M: mean; SD: standard deviation.

	All (n = 74)	EAERR (n = 30)	EAEGT (n = 23)	WN (n = 21)
Gender	Male: 48 (62%)	Male: 20 (67%)	Male: 13 (57%)	Male: 15 (71%)
	Fem: 26 (38%)	Fem: 10 (33%)	Fem: 10 (43%)	Fem: 6 (29%)
Age (years)	M: 52.9 SD: 11.8	M: 53.3 SD: 12.2	M: 51.9 SD. 11.7	M: 53.3 SD: 11.0
Tinnitus duration (years)	M: 7.8 SD: 10.8	M: 8.2 SD: 11.5	M: 9.1 SD: 10.7	M: 5.8 SD: 10.2
Tinnitus pitch (Hz)	M: 4342 SD: 2563	M: 5242 SD: 2888	M: 3344 SD: 2113	M: 4149 SD: 2140
THI_baseline_	M: 53.5 SD: 22.2	M: 50.85 SD: 23.2	M: 52.9 SD: 21.0	M: 58.8 SD: 22.6

**Table 2:** Number of non-responding subjects in each sound therapy group along with the gender, age, tinnitus duration, tinnitus frequency, and initial THI score. M: mean; SD: standard deviation.

	All (n = 18)	EAERR (n = 7)	EAEGT (n = 5)	WN (n = 6)
Gender	Male: 16 (89%)	Male: 7 (100%)	Male: 3 (60%)	Male: 6 (100%)
	Fem: 2 (11%)	Fem: 0 (0%)	Fem: 2 (40%)	Fem: 0 (0%)
Age (years)	M: 58.1 SD: 13.3	M: 57.3 SD: 10.4	M: 58.4 SD. 15.7	M: 58.8 SD: 13.1
Tinnitus duration (years)	M: 8.7 SD: 10.6	M: 3.0 SD: 0.9	M: 6.7 SD: 6.8	M: 17.0 SD: 12.9
Tinnitus pitch (Hz)	M: 5762.6 SD: 3414.2	M: 5702.7 SD: 2453.6	M: 5947.0 SD: 5345.0	M: 5678.7 SD: 1468.8
THI_baseline_	M: 53.7 SD: 23.2	M: 52.3 SD: 24.2	M: 58.8 SD: 23.4	M: 51.0 SD: 18.7

**Table 3:** Mean values of the initial, final, and change (initial–final) scores for the THI, TFI, and HADS questionnaires of the responding participants.

Stimuli	THI			TFI			HADA			HADD		
	THI_0_	THI_4_	ΔTHI	TFI_0_	TFI_4_	ΔTFI	HADSA_0_	HADSA_4_	ΔHADSA	HADSD_0_	HADSD_4_	ΔHADSD
EAERR	50.8	24.7	26.1 (***)	51.8	25.5	26.2 (***)	11.4	5.9	5.5 (**)	6.6	4.1	2.5 (*)
EAEGT	52.9	25.9	27.0 (***)	55.2	27.7	27.4 (***)	10.6	5.7	4.9 (***)	6.9	3.5	3.4 (***)
WN	58.0	29.1	28.9 (***)	60.2	31.1	29.2 (***)	10.1	6.5	3.6 (**)	6.7	4.3	2.4

* 0.01 < *p* < 0.05; ** 0.001 < *p* < 0.01; *** *p* < 0.001.

**Table 4:** Mean values of the initial, final, and change (initial–final) scores for the THI, TFI, and HADS questionnaires of the non-responding participants.

Stimuli	THI			TFI			HADA			HADD		
	THI_0_	THI_4_	ΔTHI	TFI_0_	TFI_4_	ΔTFI	HADSA_0_	HADSA_4_	ΔHADSA	HADSD_0_	HADSD_4_	ΔHADSD
EAERR	52.2	51.4	0.9	57.3	56.4	0.9	10.9	9.0	1.9	6.9	4.9	2.0
EAEGT	58.8	64.0	−5.2	61.4	63.9	−2.6	10.8	11.2	−0.4	8.0	9.6	−1.6
WN	51.0	63.0	−12.	58.3	73.5	−15.3	7.8	9.3	−1.5	5.5	7.0	−1.5

## Data Availability

The data are not publicly available due to the confidentially clause of the informed consent form.
